# Supplementary material for: The causal relationship between the human gut microbiota and pyogenic arthritis: a Mendelian randomization study
Source: Front Cell Infect Microbiol. 2024 Nov 26;14:1452480. doi: 10.3389/fcimb.2024.1452480 (PMC11629706; doi:10.3389/fcimb.2024.1452480)
Supplement: Supplementary file 1 [file DataSheet1.zip › mendelian test/ebi-a-GCST90027766.csv_leave_one_out.pdf]

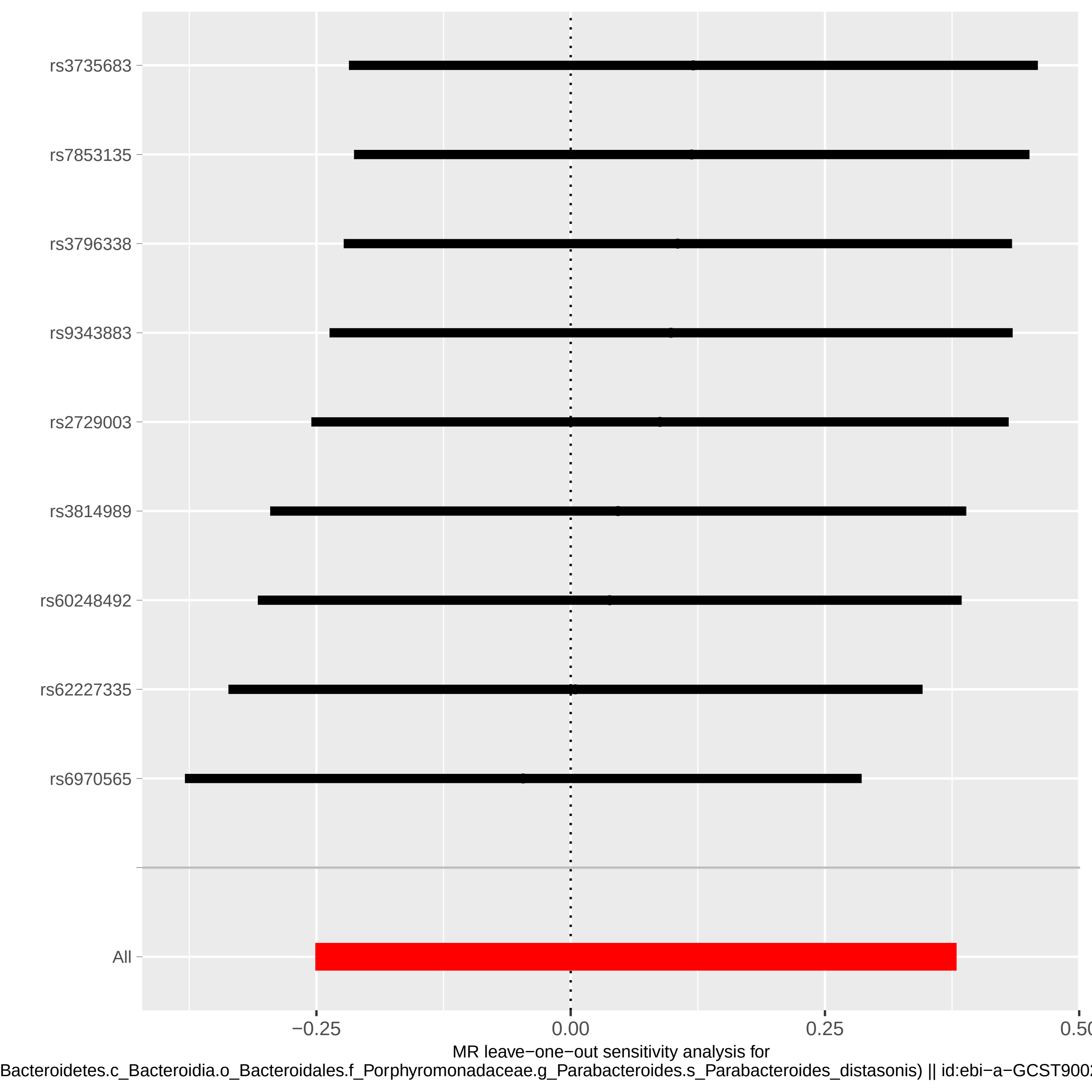

rs3735683
rs7853135
rs3796338
rs9343883
rs2729003
rs3814989
rs60248492
rs62227335
rs6970565
All
−0.25
0.00
0.25
0.50
MR leave−one−out sensitivity analysis for
Bacteroidetes.c_Bacteroidia.o_Bacteroidales.f_Porphyromonadaceae.g_Parabacteroides.s_Parabacteroides_distasonis) || id:ebi−a−GCST900
